# The use of the detailed fetal anatomy ultrasound in the first and second trimesters

**DOI:** 10.1002/pmf2.70133

**Published:** 2025-10-13

**Authors:** Lauren C. London, Anthony C. Sciscione

**Affiliations:** ^1^ Department of Obstetrics and Gynecology Christiana Care Health Services Newark Delaware USA; ^2^ Delaware Center for Maternal Fetal Medicine Newark Delaware USA

The use of diagnostic ultrasound to detect congenital anomalies and genetic syndromes has become embedded into modern prenatal care in many health systems including the United States. Traditionally, the use of sonography for the detection of genetic syndromes and congenital anomalies has been confined to the second and third trimesters as a Detailed Second Trimester Ultrasound (DSTU). With the advent of more advanced technology and the specialization of technical skills, the ability to detect many anomalies has moved to the first trimester, called the Detailed First Trimester Ultrasound (DFTU). This is highly valued by pregnant patients for a myriad of reasons, including that it can allow for earlier access to specialized prenatal resources and potentially more time for decisions on pregnancy continuation.

In 2020, the American Institute of Ultrasound in Medicine (AIUM) released a practice parameter for the performance of the DFTU. This document includes the indications, standards for performance, and training requirements for the performance of the DFTU. In clinical practice, there have been two practice paradigms for the application of the DFTU: first, and most commonly used in those performing the DFTU, is the use of the DFTU in high‐risk individuals as defined by the 2020 AIUM practice parameters; second is the use in all patients regardless of risk.

The current practice pattern described by AIUM, with endorsement from multiple other professional societies, recommends a DFTU following AIUM practice parameters in “high risk” patients as defined by any of 11 indications [1].

However, in the interim since the 2020 AIUM‐led DFTU practice parameter publication, multiple reports have emerged supporting the accuracy of the DFTU in detecting congenital anomalies in the general population, strongly suggesting that the DFTU, following the AIUM practice parameters and performed by accredited sonographers, should be offered to all patients regardless of risk factors [2, 3, 4]. Recent reports have found that the DFTU is a reliable tool for early anomaly detection, with a 50% or more detection rate for significant structural malformations in the low‐risk population [1, 3, 4, 5]. In a recent retrospective report of performing the DFTU on “all comers,” the rate of finding an ultrasound anomaly was approximately 1 in 90, with approximately 52% having low‐risk cell‐free fetal DNA (cffDNA) results [6]. This supports prior work suggesting that about 5% of patients with low‐risk cffDNA may still have anomalies identified on DFTU [7]. The DFTU has been shown to have an overall low false positive rate [3], with higher sensitivity when a formalized anatomic protocol such as the one presented by the AIUM is implemented [4, 5]. The use of only a basic fetal anatomic assessment in the second trimester may miss a substantial proportion of fetal anomalies [8], and current literature suggests that implementation of a DFTU followed by a DSTU may improve detection rates by up to 33% compared to either alone [9].

However, a discussion regarding the implementation of the DFTU to all patients would not be complete without acknowledging the practical tools required to do so, including the cost of implementation and the ability to bill insurance companies for the provided service.

From a health care cost standpoint, current literature suggests that the use of the DFTU may be a cost‐effective change when implemented universally [4]. We acknowledge the potentially large upfront costs associated with universal implementation of the DFTU—specifically, the need for high‐level sonographer training and accreditation, as well as potentially increased scanning time [4]. These costs, however, may be somewhat offset by the increase in maternal quality‐adjusted life‐years (QALYs) gained by the reassurance associated with a low‐risk DFTU and the decreased cost associated with potentially earlier termination of pregnancy in those who elect to pursue that (and therefore, the loss of cost associated with raising a child with a major congenital anomaly) [4]. The latter situation would affect a small proportion of people receiving the DFTU, and as such, the universal adoption of the DFTU is likely to increase the overall prenatal diagnosis health care cost per patient. In the context of the United Kingdom's health care system, this cost was approximated to be as low as 11 euros, or about $12, per person [4]. By conservative estimates, society's maximum willingness to pay for the small but significant increase in QALYs associated with universal DFTU means that universal implementation of DFTU is up to 95.5% likely to be cost‐effective from this perspective [4]. However, further research into the cost effectiveness of the DFTU within the health care system of the United States is needed.

From an insurance standpoint, there may be a new opportunity for providers and patients to achieve insurance coverage for the DFTU. Initially, with the publication of the AIUM DFTU practice parameters, there was not a billing code or sustainable billing path associated with its performance. The Society for Maternal‐Fetal Medicine coding committee provided billing advice, but, in the absence of a formalized CPT^®^ Code for the DFTU, they recommended “work around” codes where providers would combine several already established CPT^®^ Codes with the goal of expressing the adequate level of care provided. In response to this billing quandary, a multi‐society document was recently published that allows for the use of CPT^®^ Code 76811 twice in a pregnancy [10]. The 76811 billing code is defined as “ultrasound, pregnant uterus, real time with image documentation, fetal and maternal evaluation plus detailed fetal anatomic examination, transabdominal approach, single or first gestation” and has historically been limited to one use per pregnancy [10]. Most commonly, high‐risk patients would receive a DSTU utilizing this code. This landmark, multi‐agency recommendation to utilize Code 76811 twice in a single pregnancy was based on new and emerging evidence about the use of the DFTU to help detect genetic abnormalities and congenital anomalies.

We acknowledge that the implementation of the DFTU in routine screening may present logistical challenges, especially with the degree of expertise and training that is needed to assure a high level of screen performance. The DFTU should be performed by accredited sonographers following the AIUM practice parameters for optimal performance, which may be a challenge for many locations where access to trained sonographers and physicians is already limited. Maternity care deserts already contribute to significant disparities in maternal and child outcomes [11], and up to one‐third of counties in the United States are comprehensive reproductive health care deserts [12]. That being said, in an era when access to care may be limited by social, political, and financial factors, early anomaly detection is important to the wholistic well‐being of many patients. Near universal adoption of the DFTU may allow for more equitable access to reproductive decision‐making resources, especially when these resources may have associated geographical barriers [2]. At this time, it is unclear what the effect of universal implementation of the DFTU would be on our already overextended maternal health care system as a whole, as well as on maternal health disparities, and how this may be offset by the increased availability of reproductive decisions provided by earlier anomaly detection.

In conclusion, we believe that the universal implementation of the DFTU holds promise, giving patients earlier access to information that is potentially critical for pregnancy decisions. While more research is needed surrounding this specific technology, the advent of proper billing removes another potential impediment to the use of the DFTU in the pregnant population. We are excited about the possibilities this new tool holds for the prenatal diagnostic sector of maternal care.

## CONFLICT OF INTEREST STATEMENT

The authors declare no conflicts of interest.

## FUNDING INFORMATION

None.
